# Effect of Geometrical Configuration and Strain Rate on Aluminum Alloy 5083 and S550 Steel Characterized by Digital Image Correlation

**DOI:** 10.3390/s25123607

**Published:** 2025-06-08

**Authors:** Cheng Chen, Liuyang Feng

**Affiliations:** 1 Department of Civil and Environmental Engineering, Centre for Offshore Research and Engineering, National University of Singapore, Singapore 117576, Singapore; cccee@nus.edu.sg; 2 School of Aeronautics, Northwestern Polytechnical University, Xi’an 710072, China

**Keywords:** strain, strain rate, fracture, digital image correlation, coupon test, split-Hopkinson bar

## Abstract

This manuscript proposes a non-contact approach to characterize geometrical configuration and strain rate effects using digital image correlation (DIC). The non-contact DIC technique allows more robust and accurate material property assessment than conventional in-contact gauges, especially under dynamic loading conditions. This study first demonstrates DIC-based strain measuring accuracy in quasi-static coupon tests with two geometrical configurations. In comparison to the conventional method, DIC measures a wider range of strain up to the final fracture while eliminating geometric constraints typically imposed on test specimens. This study further extends the DIC measurement in dynamic material property tests, i.e., the split-Hopkinson bar test. The direct strain measurement by DIC presents enhanced accuracy compared to the conventional method, as the latter overestimates the strain results from remotely installed strain gauges. The deformation analysis explains the discrepancy in strain measurement at different sensor locations. The strain rate effects on the stress–strain curve and fracture strain are evaluated on two materials, i.e., aluminum alloy 5083 and S550 steel. The proposed DIC approach enables more convenient and robust measurement of strains, which facilitates the material property evaluation under various geometrical configurations and strain rates.

## 1. Introduction

Digital image correlation (DIC) is a widely adopted non-contact optical technique for convenient full-field displacement and strain measurement [1,2,3,4]. By tracking the motion of a random speckle pattern applied to a specimen surface across a sequence of images, DIC enables accurate reconstruction of displacement and strain maps [5,6]. Compared to traditional point-based measurement methods such as extensometers and strain gauges, DIC provides full-field strain measurements with high spatial resolution and therefore offers significant advantages, especially dealing with complex specimens or challenging testing environments [7]. The effectiveness and accuracy of DIC techniques in strain measurements have been widely validated under various static loading scenarios such as tension [8,9], compression [10,11], and bending [12,13]. Furthermore, DIC allows the assessment of elastic–plastic mechanical parameters (Young’s modulus, yield strength, and ultimate tensile strength [14,15,16,17,18]) and failure-related behaviors (strain localization, necking, and damage initiation [19,20,21]). The integration of DIC with finite element methods has extended its application into three-dimensional stress–strain analysis, enabling more comprehensive investigation of the internal deformation and fracture mechanisms [22,23,24]. The real-time monitoring of DIC facilitates the investigation of the loading ratio [25,26], loading rate [27] on the material responses, as well as the fracture toughness assessment [28,29,30].

The split-Hopkinson bar system has been widely utilized for characterizing material behaviors under high-strain-rate loading conditions [31,32]. The split-Hopkinson bar system includes various loading types, including tension, pressure, torsion, bending, and combined loading conditions [33,34,35,36,37]. The split-Hopkinson bar system typically comprises an incident bar and a transmitter bar, with the test specimen positioned between them. The strain and stress of the specimen are measured from strain gauges adhered remotely to the incident and transmitter bars under classical wave theories [38]. This indirect measurement is susceptible to effects such as wave dispersion and oscillation, leading to errors in the strain and elastic modulus assessment [39,40]. Verleysen et al. investigate the influence of specimen geometry on the split-Hopkinson bar test results. The authors identify that the size of the transition zone has significant effects on the strain measuring accuracy [41]. Huh et al. report the underestimation of elastic modulus in the dynamic stress–strain curves measured from the split-Hopkinson bar tests [42]. Gama et al. recommend general setup of the bar configuration and the strain gauge position to alleviate the measuring errors in the impact tests [43]. Several studies have recommended dog-bone specimens with higher effective length-to-width ratio (≥1) to obtain more accurate strain results [41,44,45]. Although recommendations have been proposed for optimizing bar configurations and specimen geometries, the accuracy of strain measurements remains uncertain due to the inherent limitations of these indirect methodologies, which leads to biased assessments of material properties.

The integration of high-speed DIC into split-Hopkinson bar experiments enables direct and real-time measurement of full-field surface strain on specimens during dynamic loadings [46]. Several studies have demonstrated the effectiveness of dynamic strain measurement using DIC [47,48,49,50]. However, to the authors’ knowledge, the discrepancy between the direct (via DIC) and indirect (via strain gauge) strain measuring methods has not been discussed in detail, and the rationale behind such discrepancies has not been fully understood either. To investigate the inherent limitation and quantify the errors from traditional approach, this study compares the strain measurement from indirect strain gauge approach and direct DIC approach under both quasi-static and dynamic loading conditions. The virtual extensometers, defined in the DIC-measured displacement field, enabling direct assessment of strain from various locations rather than a single output by traditional physical gauges. Firstly, the quasi-static coupon tests are conducted to demonstrate the efficacy of DIC in measuring the material property such as elastic modulus, Poisson’s ratio, fracture strain, and stress–strain curves. Secondly, the split-Hopkinson bar specimens are loaded under quasi-static conditions to investigate the geometry effects on the material properties. Finally, the split-Hopkinson bar tests are performed to identify the strain measuring errors from direct and indirect methods. Two material types, i.e., the aluminum alloy 5083 (AA5083) and S550 steel, are tested in this study to confirm the enhanced strain measuring accuracy of the proposed DIC approach. Through the full-field measurement, DIC allows direct quantification of the geometrical configuration and strain rate effects on the material stress–strain curve, fracture mode, and the fracture strains.

## 2. Methodology

**Figure 1:**
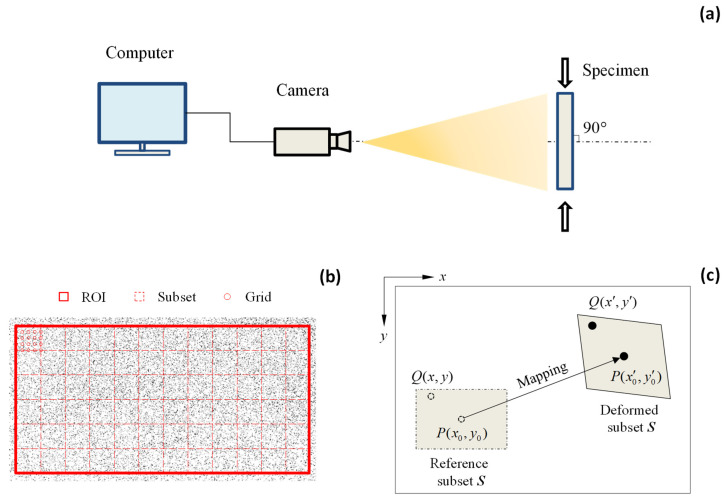
(**a**) DIC system; (**b**) definition of ROI, subset, and grid; and (**c**) the displacement mapping.

Digital image correlation (DIC) is a non-contact optical technique grounded in computer vision and has been widely employed for full-field displacement and strain measurements. Figure 1a depicts a conventional image acquisition setup used in two-dimensional DIC applications. In this configuration, a high-resolution camera is oriented orthogonally to the specimen surface to ensure accurate imaging. To enhance the image fidelity, the optical axis of the camera must remain parallel to the specimen plane. Key imaging parameters such as aperture, depth of field, and exposure time must also be carefully optimized to ensure the quality and reliability of the DIC results.

The correlations of DIC images are performed through computer-aided processing software. Since the processing of the entire image is computationally intensive and often unnecessary, DIC analysis is typically restricted to a designated area known as the region of interest (ROI). As illustrated in Figure 1b, the ROI is subdivided into smaller sections called subsets, which are arranged according to a pre-defined grid spacing. Each subset contains a distinct speckle pattern that serves as a reference for displacement tracking. The grid spacing represents the interval between adjacent evaluation points within the subset. The selection of ROI dimension, subset size, and grid spacing is influenced by the quality and density of the speckle pattern, as well as the computational resources available.

Once the image has been discretized, each subset is individually analyzed to generate a displacement map. Figure 1c illustrates the deformation of an arbitrary subset from its original (reference) configuration to its deformed state. Let point $P(x_{0},y_{0})$ represent the center of the subset domain ***S***. To determine the corresponding position of $P(x_{0}^{\prime },y_{0}^{\prime })$ in the deformed image, the pixel intensity values within subset ***S*** are extracted and organized into a matrix that characterizes its unique grayscale distribution. This reference matrix is then systematically compared against candidate matrices in the deformed image using cross-correlation algorithms. There are several commonly used criteria for identifying the correlation process, i.e., cross-correlation (CC), normalized cross-correlation (NCC), zero-normalized cross-correlation (ZNCC), sum of squared difference (SSD), normalized sum of squared difference (NSSD), and zero-normalized sum of squared difference (ZNSSD). Pan et al. has demonstrated enhanced robustness of ZNCC and ZNSSD against varying lighting conditions [51]. Similar recommendations are also reported by Tong [52]. Therefore, this study employs the zero-normalized sum of squared difference (ZNSSD) criterion with the following mathematical formulation:

(1) $$C={\sum_{i,j\in S}\left[\frac{f(x_{i},y_{j})-f_{m}}{\Delta f}-\frac{g(x_{i}^{\prime },y_{j}^{\prime })-g_{m}}{\Delta g}\right]}^{2}$$

(2) $$\Delta f=\sqrt{{\sum_{i,j\in S}\left[f(x_{i},y_{j})-f_{m}\right]}^{2}}$$

(3) $$\Delta g=\sqrt{{\sum_{i,j\in S}\left[g(x_{i}^{\prime },y_{j}^{\prime })-g_{m}\right]}^{2}}$$

where f and g are the pixel intensity under reference and deformed configurations, $f_{m}$ and $g_{m}$ denote the mean intensity value, and Δf and Δg represent the corresponding variations.

With the assumption of continuous deformation, all internal grid points in a reference subset follow the same mapping rule. Point $Q(x_{i},y_{j})$ is an arbitrary point inside the reference subset ***S***. According to the mapping function, the following equations are established to track Point *Q* under deformation configurations,

(4) $$x_{i}^{\prime }=x_{i}+\xi (x_{i},y_{j})$$

(5) $$y_{j}^{\prime }=y_{j}+\eta (x_{i},y_{j})$$

where ξ and η denote the shape functions, i,j∈S. The formulation of shape functions relies on a Taylor series approximation to ensure accurate interpolation within the domain. A zero-order shape function is only capable of depicting the rigid-body motion, while the first-order shape function allows tracking of translation, rotation, shear, and combinations [53]. This study employs second-order shape function to obtain enhanced measuring accuracy under large deformations [54]. Higher-order terms beyond the second order have limited impact on accuracy while significantly increasing computational cost and are therefore inefficient for the purposes of this study [55]. The second-order shape function follows,

(6) $$\begin{array}{ll} \xi (x_{i},y_{j})= & u_{x}+\frac{\partial u_{x}}{\partial x}(x_{i}-x_{0})+\frac{\partial u_{x}}{\partial y}(y_{j}-y_{0})+\frac{1}{2}\frac{\partial^{2}u_{x}}{\partial x^{2}}(x_{i}-x_{0}{)}^{2}+\frac{1}{2}\frac{\partial^{2}u_{x}}{\partial y^{2}}(y_{j}-y_{0}{)}^{2}+\\ & \frac{1}{2}\frac{\partial^{2}u_{x}}{\partial x\partial y}(x_{i}-x_{0})(y_{j}-y_{0}) \end{array}$$

(7) $$\begin{array}{ll} \eta (x_{i},y_{j})= & u_{y}+\frac{\partial u_{y}}{\partial x}(x_{i}-x_{0})+\frac{\partial u_{y}}{\partial y}(y_{j}-y_{0})+\frac{1}{2}\frac{\partial^{2}u_{y}}{\partial x^{2}}(x_{i}-x_{0}{)}^{2}+\frac{1}{2}\frac{\partial^{2}u_{y}}{\partial y^{2}}(y_{j}-y_{0}{)}^{2}+\\ & \frac{1}{2}\frac{\partial^{2}u_{y}}{\partial x\partial y}(x_{i}-x_{0})(y_{j}-y_{0}) \end{array}$$

where $u_{x}$ and $u_{y}$ represent the horizontal displacement and the vertical displacement at point *P*, respectively. The Newton–Raphson optimization method greatly improves the efficiency of displacement mapping.

**Figure 2:**
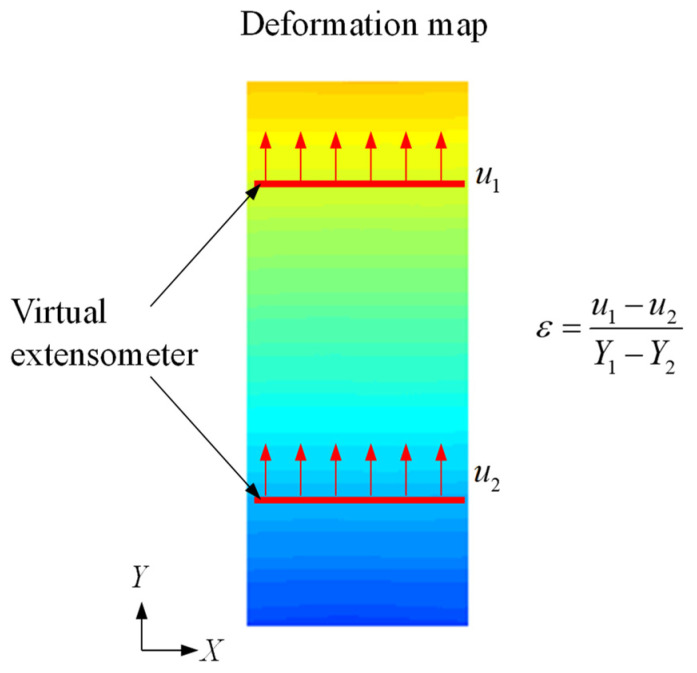
Definition of the virtual extensometer (VE) in DIC.

This study determines the strain from a virtual extensometer (VE) defined in DIC,

(8) $$\varepsilon =\frac{u_{1}-u_{2}}{Y_{1}-Y_{2}}$$

where $u_{1}$ and $u_{2}$ measure the elongation of the virtual extensometer between $Y_{1}$ and $Y_{2}$. Figure 2 illustrates the definition of a virtual extensometer in DIC-measured displacement field. In comparison to the conventional pointwise strain measurement, DIC allows versatile definitions of the virtual extensometers, which characterize the material responses from different sensor locations.

## 3. Quasi-Static Coupon Tests

This section elucidates the coupon test under quasi-static loading conditions of two materials, i.e., aluminum alloy 5083 (AA5083) and S550 steel. Two types of coupon specimens are considered with different geometrical configurations, as summarized in Table 1. The large coupon specimen follows the geometrical configuration as suggested by ASTM E8/E8M [56], while the small coupon specimen is designed for the split-Hopkinson bar tests under dynamic loadings. The material properties are evaluated under two different configurations to investigate the effects of specimen geometry.

### 3.1. Large Coupon Specimens

**Figure 3:**
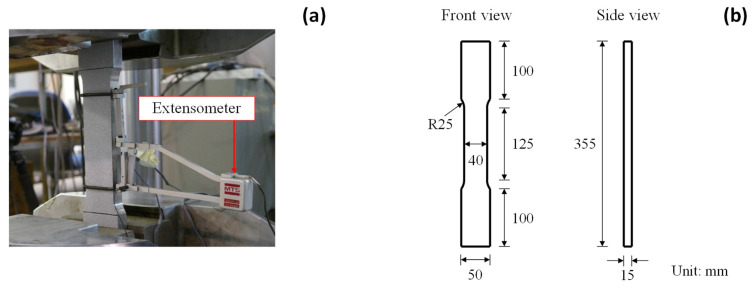
Large coupon specimen: (**a**) test setup and (**b**) geometrical configuration.

Figure 3a presents the test setup of the large coupon specimens. The specimen has a total length of 355 mm and thickness of 15 mm, as shown in Figure 3b. Following the recommendation in ASTM E8/E8M [56], the gauge area of the large coupon specimen is set as $125\times 40{\;\mathrm{mm}}^{2}$. The coupon test is conducted under a universal MTS machine, which has a maximum load of 1000 kN. Two ends of the large coupon specimen are clamped by the MTS machine and subjected to the displacement-controlled loading at a rate of 0.3 mm/min. The physical extensometer has a gauge size of 100 mm and is attached to the specimen’s gauge area to measure the elongation, as indicated in Figure 3a.

**Figure 4:**
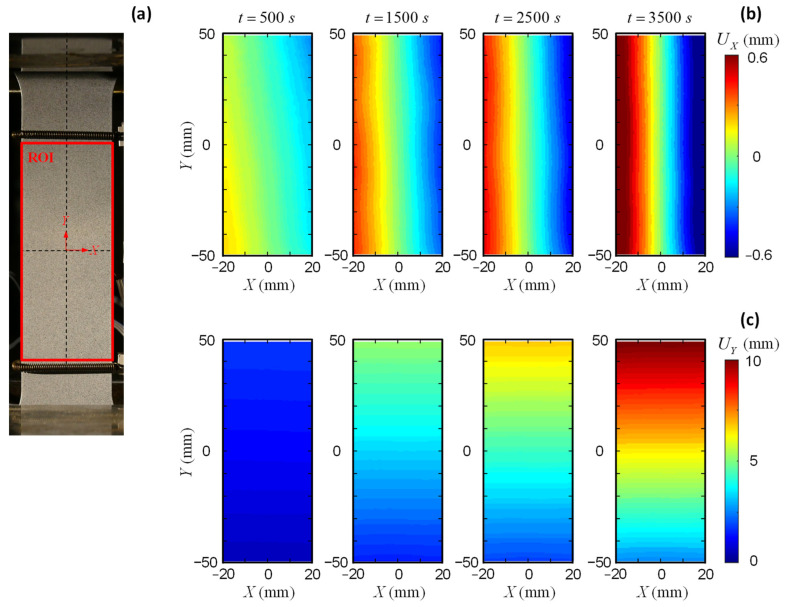
Large coupon AA5083 specimen: (**a**) ROI definition, (**b**) $U_{X}$ field, and (**c**) $U_{Y}$ field.

In contrast to the physical extensometer, this study defines several virtual extensometers (VEs) in DIC to measure the strain from different ranges. The full-field DIC measurement allows versatile VE definitions to compare the strain measuring accuracy. Figure 4a illustrates the image captured by the DIC camera with a high resolution of 6016×4016 pixels. A region of interest (ROI) is defined between the strings of the physical extensometer, roughly covering a range of $40\times 100{\;\mathrm{mm}}^{2}$. The origin of the global coordinates is located in the middle of the defined ROI. The DIC images are processed in the commercial software VIC-2D [57]. A subset of $1\times 1{\;\mathrm{mm}}^{2}$ is adopted for the displacement mapping. The current study only involves displacement output from DIC, which avoids the displacement-to-strain derivation that requires additional noise-filtering procedures. The strain of the coupon specimen is derived from Equation (8) based on elongation instead of direct pointwise output.

**Figure 5:**
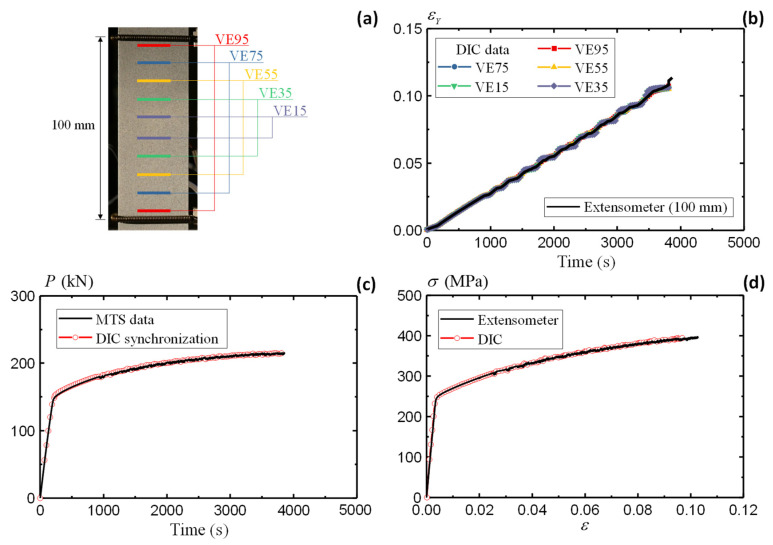
Large coupon AA5083 specimen: (**a**) definition of virtual extensometer, (**b**) strain comparison, (**c**) load synchronization, and (**d**) stress–strain curve comparison.

Figure 4b,c illustrates the evolution of the $U_{X}$ and $U_{Y}$ fields of the AA5083 specimen during the loading process. The contraction in the *X* direction and the elongation in the *Y* direction are accurately captured by the DIC measurements. Figure 5a presents the VE definitions along the *Y* direction. Five VEs are defined within the ROI, i.e., VE95, VE75, VE55, VE35, and VE15. Similar to a physical extensometer, each VE consists of two ends symmetrically positioned relative to the *X* axis (Figure 2). Each end comprises a single layer of nodes at corresponding positions. The displacement values are averaged over the defined layer to derive the strain using Equation (8). Figure 5b summarizes the strain output from the VEs at varying locations. The strain measurements obtained from different VEs show good agreement, indicating uniform elongation within the gauge section. The results from the DIC-based VEs are also consistent with the data recordings from the physical extensometer. A fracture strain of 0.12 is observed from the strain measurement in Figure 5b.

Stress is the other essential output to evaluate the material property. This study determines the DIC stress output through synchronizing DIC with the machine load responses. With the synchronized setup, the load output for DIC is determined from the MTS recorded data, as shown in Figure 5c. The synchronized DIC load agrees well with the original MTS data, demonstrating the effectiveness of the camera triggering system. With the strain and stress obtained separately, Figure 5d summarizes the true stress–strain curve using DIC and extensometer methods. The coinciding curves demonstrate the accuracy of the non-contact DIC approach in evaluating the constitutive laws for a given material.

**Figure 6:**
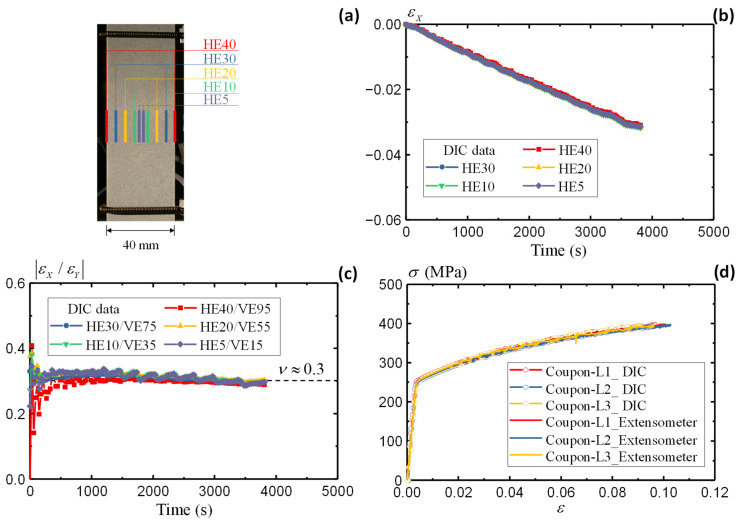
Large coupon AA5083 specimen: (**a**) definition of horizontal extensometer, (**b**) strain comparison, (**c**) Poisson’s ratio, and (**d**) summary of stress–strain curves.

Besides the tensile strain measurement along the vertical direction (elongation), DIC also enables the compressive strain evaluation along the horizontal direction (contraction). Such a combination helps to evaluate the Poisson’s ratio. Figure 6a defines the virtual extensometer along the horizontal direction (HE) in five ranges, i.e., HE40, HE30, HE20, HE10, and HE5. Figure 6b summarizes the horizontal strain from different HEs. Negative values are present due to the compressive strain along the *X* direction. The agreeable results from different locations indicate uniform contraction of the specimen. In conjunction with the vertical strain in Figure 5, the Poisson’s ratio is determined by

(9) $$\nu =\left|\frac{\varepsilon_{X}}{\varepsilon_{Y}}\right|$$

Figure 6c illustrates the variation in the derived Poisson’s ratio during the test. The comparison starts from a relatively large range at HE40/VE95 and gradually approaches the center area at HE5/VE15. The initial scatters indicate large noises when the strains are not pronounced in both directions. The $\varepsilon_{X}/\varepsilon_{Y}$ results converge after 500 s when all five pairs of HE/VEs present consistent values, oscillating slightly around 0.3. Therefore, the Poisson’s ratio is determined as 0.3 for the AA5083 material.

Following the same procedures, Figure 6d summarizes the stress–strain relationship of all three large coupon specimens measured by the DIC method. The comparison with conventional in-contact methods demonstrates the accuracy and robustness of the DIC approach in assessing the material properties. All three specimens present consistent material responses under quasi-static loading conditions.

### 3.2. Small Coupon Specimens

**Figure 7:**
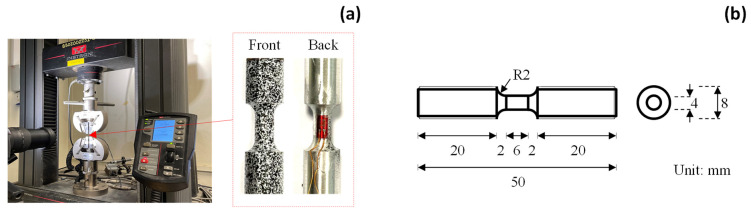
Small coupon specimen: (**a**) test setup and (**b**) geometrical configuration.

Figure 7a presents the test setup of the small coupon specimens. In line with the split-Hopkinson bar tests, the small coupon tests employ rod specimens, with the configuration presented in Figure 7b. The specimen has a total length of 50 mm and a global diameter of 8 mm. The length and diameter of the gauge area are 6 mm and 4 mm, respectively. The length-to-width ratio is 1.5, following the recommendation in [41]. The small coupon tests are performed under an Instron machine with a maximum load of 50 kN. The Instron machine applies a displacement-controlled loading at a rate of 0.1 mm/min by clamping both ends of the specimen.

**Figure 8:**
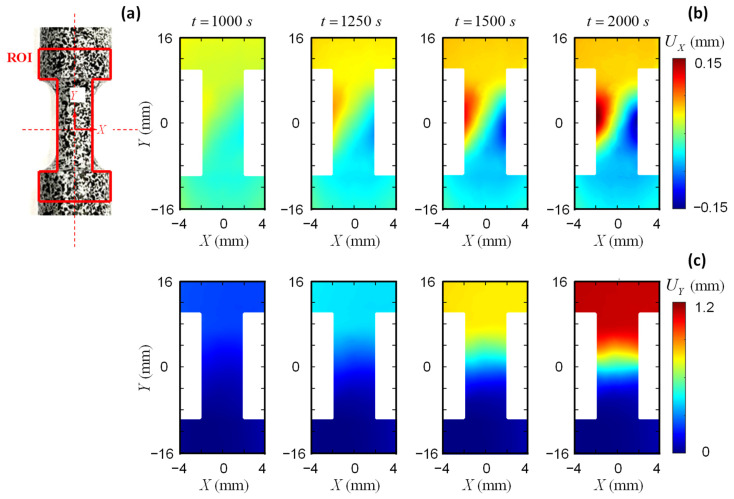
Small coupon AA5083 specimen: (**a**) ROI definition, (**b**) $U_{X}$ field, and (**c**) $U_{Y}$ field.

The front surface of the small coupon specimen is covered with speckles for DIC images, while the back surface attaches a strain gauge at the center of the gauge area to measure the strain (Figure 7a). The high-resolution camera is employed to record DIC images during the tensile loading. Figure 8 illustrates the elongations of the small coupon specimen made of AA5083 material. The ROI has an I-shape which lies symmetrically to the center of the coupon specimen. The DIC parameters follow the same setting as the previous analyses in Section 3.1. In contrast to the large coupon specimen, the displacement map of the small coupon specimen shows localized deformation at the center of the gauge area (Y≈0), both in the *X* and *Y* directions. This indicates the necking of the dog-bone specimen, which eventually leads to the fracture of the small coupon specimen.

### 3.3. Strain Measurement Accuracy

**Figure 9:**
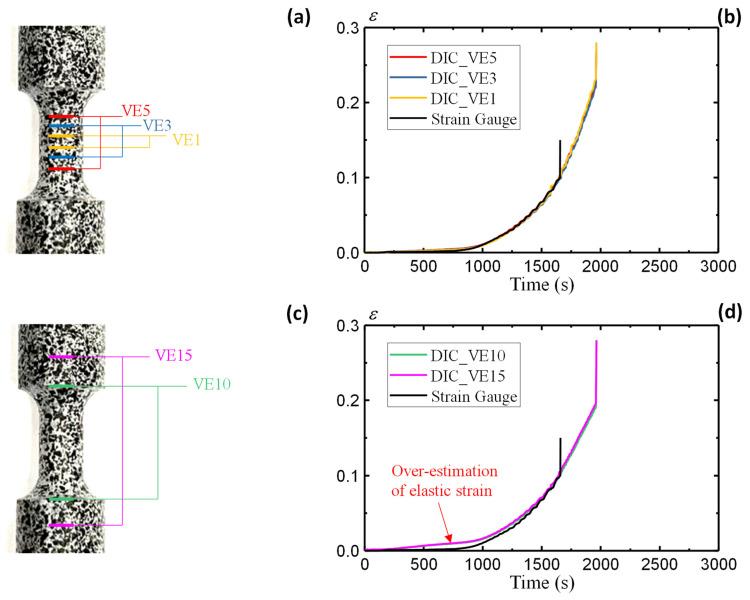
Small coupon AA5083 specimen: (**a**) definition of virtual extensometers inside the gauge area, (**b**) strain comparisons, (**c**) definition of virtual extensometers outside the gauge area, and (**d**) strain comparisons.

This section highlights the effect of sensor location on the strain measuring accuracy using the AA5083 specimen results. Two groups of virtual extensometers (VEs) are defined with varying locations, as shown in Figure 9. The first group is located inside the gauge area, with three VE definitions, namely VE5, VE3, and VE1, as shown in Figure 9a. The strain results from the three definitions (Figure 9b) remain consistent with the data measured through strain gauge. The pre-installed strain gauge, with a measuring limit of 10%, fails immediately after exceeding the measuring range. The DIC-based virtual extensometers allow continuous strain measurement without any measuring limits.

On the other hand, this section defines a second group of VEs outside the gauge area, i.e., VE10 and VE15 as shown in Figure 9c. Such VE definitions are employed to investigate the discrepancy from remote sensors that lie outside of the gauge area. For the remotely installed sensors, the strain calculation from Equation (8) employs the gauge length (6 mm) to replace the original VE length $\left(Y_{1}-Y_{2}\right)$ as suggested by [38]. This is to ensure consistent strain expression by assuming rigid-body motion outside the gauge area. Figure 9d illustrates the strain results of VE10 and VE15 and compares them with the strain gauge data. The results from VE10 and VE15 agree well with each other, demonstrating the consistence of strain measurement outside the gauge area. However, the comparison with the strain gauge data reveals significant discrepancies in the elastic stage, where VE10 and VE15 overestimate the elastic strains. The deviations decrease gradually with increasing plastic strain. The comparison of different VE results highlights the effect of sensor locations on the strain measuring accuracy. For both physical and virtual sensors, those located within the gauge area have the best measuring accuracy and are therefore recommended for material property assessments. Figure 9b observes a fracture strain of 0.22 for the small coupon specimen, which is larger than the large coupon specimen in Figure 5b.

**Figure 10:**
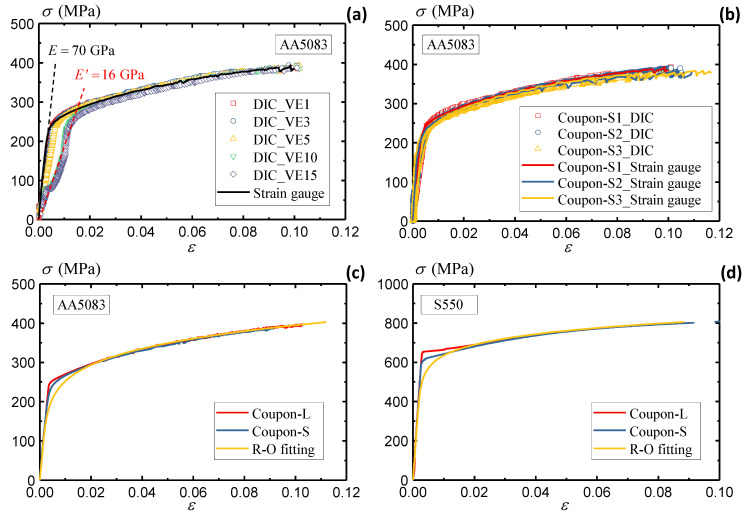
(**a**) Summary of AA5083 stress–strain curves, (**b**) comparison of DIC and strain gauge measurements of AA5083 material, (**c**) comparison of large and small AA5083 coupon specimens, and (**d**) comparison of large and small S550 coupon specimens.

Figure 10a summarizes the stress–strain curves measured from different VEs and physical strain gauge. The derivation of stress data follows a similar synchronizing method as elaborated in the previous section. Due to the overestimated elastic strain, the curves of VE10 and VE15 anticipate an underestimated elastic modulus, with only 16 GPa compared to the actual 70 GPa. The rest of the VEs defined inside the gauge area (VE1, VE3, and VE5) present agreeable stress–strain curves with the conventional methods. In conclusion, the strain measurement inside the gauge area presents the best accuracy of the material strain responses, while the measurement outside the gauge area may overestimate the strain and lead to severe discrepancies. Figure 10a summarizes the stress–strain curves from three small coupon specimens. The coinciding results measured by DIC and strain gauges demonstrate the robustness of the DIC approach in assessing the material responses. The same conclusion applies to the S550 specimens, which is not discussed in detail in this section for brevity.

### 3.4. Results Summary

Figure 10c compares the true stress–strain curve from the large and small coupon specimens of AA5083 to investigate the geometrical effects. The results from two specimen configurations, both validated by multiple specimens, present consistent responses under quasi-static loadings. There is a slight deviation near the yielding stage. The large coupon specimen observes an unremarkably higher yielding stress than the small coupon specimen. This study extracts the material constants of AA5083 following the Ramberg–Osgood equation [58],

(10) $$\frac{\varepsilon }{\varepsilon_{0}}=\frac{\sigma }{\sigma_{0}}+\alpha {\left(\frac{\sigma }{\sigma_{0}}\right)}^{n}$$

where $\sigma_{0}$ and $\varepsilon_{0}$ are the yielding stress and strain, α represents the material constant, and *n* denotes the hardening exponent. Table 2 extracts the material constants of the AA5083 material from the measured stress–strain curves. The large and small coupon specimens present consistent material constant results, as the fitted R-O curve agrees well with both curves.

Following the same procedures, Figure 10d compares the stress–strain curve of the S550 material under different specimen configurations. The insignificant deviations demonstrate consistent material properties of S550, which is also well described by the R-O fitting curve. The material constants of S550 will be presented in conjunction with the strain rate effects in Section 4 below.

## 4. Split-Hopkinson Bar Tests

**Figure 11:**
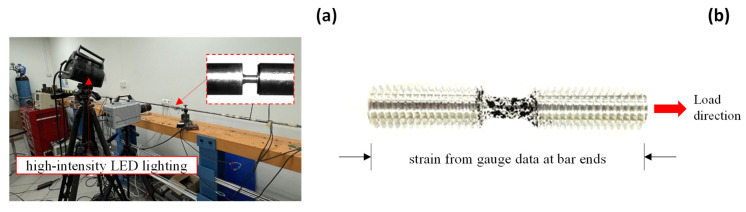
Split-Hopkinson bar specimen: (**a**) test setup and (**b**) geometrical configuration.

This section investigates the strain measurement under dynamic loading conditions. The split-Hopkinson tensile bar system is utilized to assess the AA5083 and S550 material property under increasing strain rates. Figure 11a illustrates the test setup for the split-Hopkinson bar system. The high-speed camera has a resolution of 128×64 pixels with a frame rate of 300,000 frames per second. Short exposure times are used in high-speed imaging to reduce the potential influence of motion blur or vibration-induced artifacts. High-intensity LED lighting provides stable and controlled illumination and ensures consistent image quality and contrast across all frames. The DIC camera and the Hopkinson bar are mounted separately to suppress external mechanical interference. Prior to the main experiments, a series of preliminary tests are conducted to validate the robustness and reliability of the DIC system under dynamic loading conditions. These tests ensure stable imaging performance, consistent lighting, and effective correlation accuracy, thereby minimizing the influence of environmental or system-related factors on DIC results.

Figure 11b presents the specimen configuration, which has the same size as the small coupon specimen. Both ends of the dynamic specimen are bolted with the Hopkinson bars. Two strain gauges are attached to the transmitter and incident bars. The strain and stress are derived from the following equations [59],

(11) $$\dot{\varepsilon }=2c\varepsilon_{R}/L_{s}$$

(12) $$\varepsilon =2c/L_{s}\int_{0}^{t}\varepsilon_{R}dt$$

(13) $$\sigma =EA\varepsilon_{T}/A_{s}$$

where ε and σ are the specimen strain and stress under a strain rate of $\dot{\varepsilon }$, the subscript R and T represent the reflect and transmit waves, c denotes the wave propagation velocity, $L_{s}$ and $A_{S}$ are the specimen gauge length and the cross-section area, and *E* and *A* indicate the elastic modulus and the cross-section area of the Hopkinson bars.

### 4.1. Rate Dependence of AA5083

**Figure 12:**
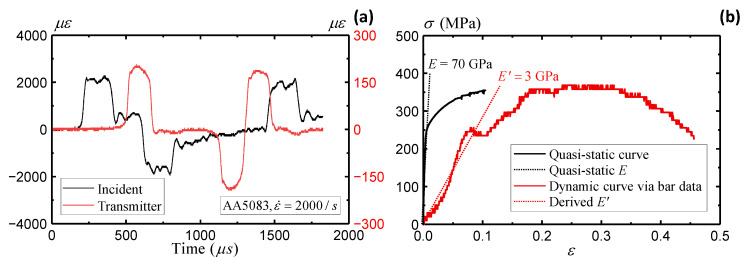
Split-Hopkinson bar AA5083 specimen: (**a**) strain gauge data and (**b**) stress–strain measurement from bar gauges.

This section evaluates the strain rate dependence of the AA5083 material. Figure 12a presents the strain readings recorded by strain gauges on the incident and transmitter bars. A strain rate of 2000/s is observed from the strain gauge data. Figure 12b derives the stress–strain curve based on Equations (11)–(13). A severe underestimation of the elastic modulus is observed, with only 3 GPa compared to the original 70 GPa. On the other hand, the yield stress is around 250 MPa, which remains close to the quasi-static yielding stress. The results indicate reasonable stress estimation from the wave theory, while the strain estimation remains questionable from the remote sensors on the Hopkinson bar ends.

**Figure 13:**
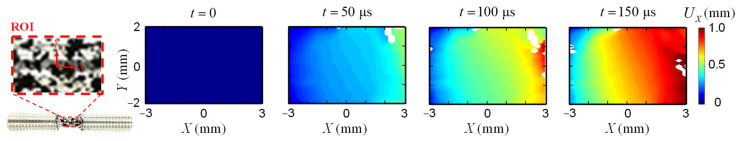
Displacement field of the split-Hopkinson bar AA5083 specimen.

**Figure 14:**
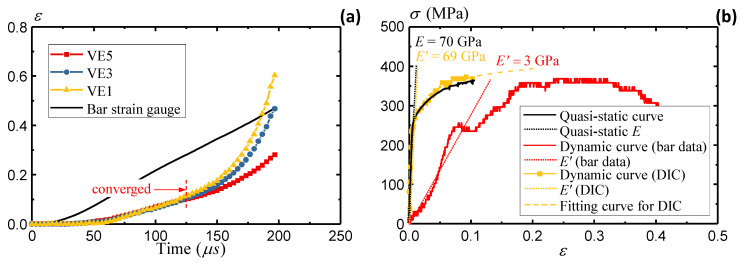
Split-Hopkinson bar AA5083 specimen: (**a**) strain comparisons and (**b**) stress–strain comparison.

Figure 13 plots the displacement field from DIC analyses. Due to the lower image quality and contrast, part of the data is missing during the high loading rates, especially near the edge of the specimen. Following the recommendation in the previous section, the virtual extensometers are defined within the gauge area to obtain more accurate strain results. Figure 14a compares the strain value through DIC and remote strain gauges. A significant overestimation of the strain is observed from strain gauges installed on the remote bars. This overestimation originates from the position of the remote sensors, as demonstrated in Figure 9c in the quasi-static tests. The derivation of strain from deformation outside the gauge section does not reflect the actual specimen deformation and therefore leads to overestimated strain values. The dynamic Hopkinson bar tests utilize sensors that are placed even further from the gauge area (at bar ends), which leads to further increased deviations. The DIC results remain consistent until 125 μs, indicating uniform elongation of the specimen. After 125 μs, the specimen presents local necking as the strain from the local VE1 is higher than the VE3 and VE5 results. The dynamic responses of the gauge area are similar to the quasi-static responses in Section 3.2. A local VE tends to enhance the strain measuring accuracy when the specimen is approaching the final failure.

Figure 14b summarizes the stress–strain curve from the DIC-measured strain data. The strain data are extracted up to 0.1 when consistent results are observed from different VEs. In comparison to the original stress–strain curve measured from remote strain gauges, DIC clearly shows more reasonable material responses by eliminating the bias in the elastic modulus. An R-O fit is applied to the DIC data to extract the material constants and alleviate the noises from dynamic loading conditions.

**Figure 15:**
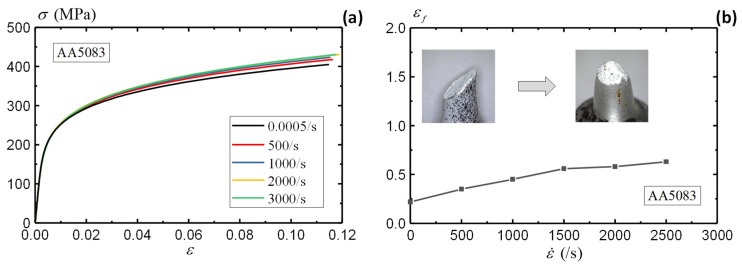
Rate dependence of AA5083 material: (**a**) stress–strain curve and (**b**) fracture strain.

Following the same procedures, Figure 15a summarizes the stress–strain curves under different strain rates using DIC-measured strains. The split-Hopkinson bar test employs 20 specimens, which cover a wide strain rate range from 500 to 3000 per second, as summarized in Table 1. The AA5083 material demonstrates insignificant rate sensitivity as the increase in the material responses is marginal under increasing strain rates. In comparison to the quasi-static condition, the stress under a strain rate of 3000/s presents only a 10% increase. The insensitivity of the AA5083 material to the strain rate is also reported in [60,61]. Therefore, the quasi-static stress–strain curve can be employed to simulate dynamic loading scenarios on a conservative basis. Figure 15b presents the fracture strains $\left(\varepsilon_{f}\right)$ of the AA5083 specimens under different strain rates. Unlike the stress–strain curves, the fracture strains of the AA5083 material demonstrate significant sensitivity to the increasing strain rates, which reflects the enhanced material ductility under higher impact rates. While the quasi-static A5083 specimen presents a shear failure mode as shown in Figure 15b, the dynamic specimens show a tensile failure mode under impact loadings. The strain rate has a clear impact on the fracture strain and failure mode, although the stress–strain curves remained similar for the AA5083 specimens.

### 4.2. Rate Dependence of S550

**Figure 16:**
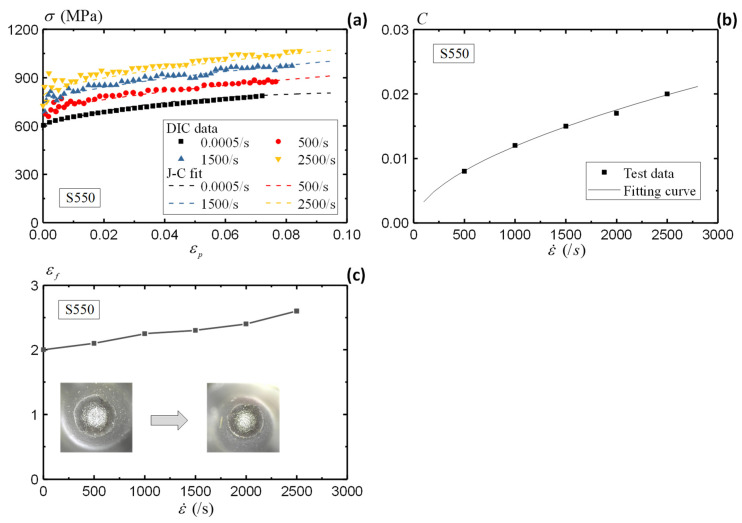
Rate dependence of S550 material: (**a**) stress–strain curve, (**b**) fitting of modified J-C model, and (**c**) fracture strain.

This section evaluates the strain rate dependence of the S550 material [62,63]. Under the same specimen configuration and test setup, Figure 16a illustrates the varying stress–strain curve of S550 under increasing strain rates. In contrast to the AA5083 material, the S550 material clearly has a higher rate sensitivity. The stress–strain responses indicate remarkable increases to the strain rates.

To quantify the strain rate dependence of the S550 material, this section utilizes a modified Johnson–Cook (J-C) model [64] with the following expression,

(14) $$\sigma =(k_{1}+k_{2}\varepsilon^{k_{3}})(1+C\ln\frac{\dot{\varepsilon }}{{\dot{\varepsilon }}_{0}})$$

(15) $$C=C_{1}{\dot{\varepsilon }}^{C_{2}}$$

where $k_{1},\;k_{2},\;k_{3}\;\mathrm{and}\;C$ are the material constants for the modified J-C model, and ${\dot{\varepsilon }}_{0}$ is the reference strain rate under quasi-static condition (0.0005/s). Figure 16b illustrates the relationship of *C* with the strain rate, which is fitted by parameters $C_{1},C_{2}$ in Equation (15). Table 3 summarizes the material constants of the S550 material. The material responses denoted by Equations (14) and (15) agree well with the test data, as shown in Figure 16a.

Figure 16c presents the fracture strains of the S550 specimens under different strain rates. The fracture strains of S550 material increases from 2.0 under quasi-static loading to 2.6 under a strain rate of 2500/s. In comparison to the AA5083 material, the S550 material presents much higher fracture resistance and material ductility. A cup-and-cone failure mode is observed from the fracture surface of the S550 specimens, both under the quasi-static and dynamic loading conditions. Therefore, there is no transition of failure mode for the S550 material. With the assistance of DIC, more comprehensive evaluations of the strain rate are presented in this study with enhanced strain measuring accuracy.

## 5. Summary and Conclusions

This study presents a DIC-based approach to enhance the accuracy of material property assessment and characterize the geometrical configuration and strain rate effects. The coupon tests under quasi-static loading conditions are employed to investigate the validity of DIC measurement through comparison with conventional methods. This study extends the DIC method in deriving the strains under dynamic loading conditions. The material properties of aluminum alloy 5083 and S550 steel are assessed to investigate the effect of geometrical configuration and strain rates. The above work supports the following conclusions:

(1) DIC enables full-field displacement measurement, which allows versatile definitions of the virtual extensometers. The virtual extensometer measures the elongation of the specimen from different ranges, which facilitates the assessment of material properties such as stress–strain curve, elastic modulus, Poisson’s ratio, and fracture strain.

(2) The strain of the coupon specimen is more accurately measured from sensors located inside the gauge area. Measurement outside the gauge area tends to overestimate the strain and leads to deviations in the elastic modulus. This phenomenon occurs under both quasi-static and dynamic loading conditions. The assistance of DIC helps to enhance the strain measuring accuracy through direct specimen deformation tracking under split-Hopkinson bar tests, while the conventional method may cause discrepancies in deriving the strain from remote sensors.

(3) The geometry of the test specimen does not affect the stress–strain curve. Both the AA5083 and S550 materials demonstrate consistent stress–strain curves under large and small coupon specimens. However, the specimen configuration poses significant influence on the fracture strains. The small rod specimen presents a much higher fracture strain than the large flat coupon specimens.

(4) The AA5083 material demonstrates insensitive rate dependence under impact loading conditions, as the dynamic stress–strain curves remain similar to those under quasi-static conditions. In contrast, the S550 material presents strong rate dependence as the stress–strain curves vary with the strain rates. This study employs a modified J-C model to describe the rate dependence of S550. Despite different rate dependence, both AA5083 and S550 materials demonstrate enhanced ductility to the strain rate, reflected by the increasing fracture strains.

## Figures and Tables

**Table 1:** Summary of test specimens.

Material	Specimen Type	Configuration (mm)	No. of Specimens	VE Ranges (mm)	Loading Condition	Strain Rate
AA5083	Large coupon	355×50×15	3	15, 35, 55, 75, 95	Quasi-static	0.0001/s
	Small coupon	Φ8×50	3	1, 3, 5, 10, 15	Quasi-static	0.0005/s
	Hopkinson	Φ8×50	20	1, 3, 5	Dynamic	500–3000/s
S550	Large coupon	355×50×15	3	15, 35, 55, 75, 95	Quasi-static	0.0001/s
	Small coupon	Φ8×50	3	1, 3, 5, 10, 15	Quasi-static	0.0005/s
	Hopkinson	Φ8×50	30	1, 3, 5	Dynamic	500–2500/s

**Table 2:** Summary of AA5083’s material properties.

E (GPa)	ν	$\sigma_{0}\;(\mathrm{MPa})$	n	α
70	0.3	258	6.0	2.0

**Table 3:** Summary of S550’s material properties.

$k_{1}\;(\mathrm{MPa})$	$k_{2}\;(\mathrm{MPa})$	$k_{3}$	$C_{1}$	$C_{2}$
600	900	0.6	0.58	0.63

## Data Availability

Data will be made available on request.
